# A Real-Time Urine Tenofovir Assay Improves Drug Adherence Among People With HIV With Prior Virologic Failure in a Randomized Controlled Trial

**DOI:** 10.1093/cid/ciaf337

**Published:** 2025-06-20

**Authors:** Gert U van Zyl, Eric Decloedt, Lauren Jennings, Tracy Kellermann, Khethiwe Motha, Marije van Schalkwyk, Chantel Schreuder, Nicola Coetzee, David V Glidden, Catherine Orrell, Monica Gandhi

**Affiliations:** Division of Medical Virology, Department of Pathology, Stellenbosch University, Cape Town, South Africa; National Health Laboratory Service, Tygerberg, Cape Town, South Africa; Division of Clinical Pharmacology, Department of Medicine, Stellenbosch University, Cape Town, South Africa; Desmond Tutu HIV Centre, Institute of Infectious Disease and Molecular Medicine and Department of Medicine, University of Cape Town, Cape Town, South Africa; Division of Clinical Pharmacology, Department of Medicine, Stellenbosch University, Cape Town, South Africa; Division of Clinical Pharmacology, Department of Medicine, Stellenbosch University, Cape Town, South Africa; Division of Infectious Diseases, Department of Medicine, Stellenbosch University, Cape Town, South Africa; Desmond Tutu HIV Centre, Institute of Infectious Disease and Molecular Medicine and Department of Medicine, University of Cape Town, Cape Town, South Africa; Division of Medical Virology, Department of Pathology, Stellenbosch University, Cape Town, South Africa; Division of HIV, Infectious Diseases, and Global Medicine, Department of Medicine, UCSF/San Francisco General Hospital, San Francisco, California, USA; Desmond Tutu HIV Centre, Institute of Infectious Disease and Molecular Medicine and Department of Medicine, University of Cape Town, Cape Town, South Africa; Division of HIV, Infectious Diseases, and Global Medicine, Department of Medicine, UCSF/San Francisco General Hospital, San Francisco, California, USA

**Keywords:** urine tenofovir, point-of-care assay, antiretroviral therapy adherence, virologic suppression, tenofovir diphosphate

## Abstract

**Background:**

Scalable strategies to detect and address inadequate adherence to antiretroviral therapy (ART) are a high priority towards meeting UNAIDS 95-95-95 targets. A urine tenofovir rapid assay (UTRA) at the point-of-care improves adherence among pre-exposure prophylaxis recipients and virologic suppression (VS) in a pre-post study of people with HIV (PWH). Here, we conducted the first randomized trial of UTRA-enhanced adherence support vs standard of care among PWH.

**Methods:**

Participants receiving dolutegravir (DTG)– or protease inhibitor (PI)–based ART were randomized to UTRA-enhanced adherence support (n = 100) vs standard of care (n = 100). The primary outcome was VS, HIV-1 RNA <50 copies/mL, at 12-months and secondary outcome was VS at 6 months. To explore ART adherence over the preceding 6–8 weeks, tenofovir diphosphate (TFV-DP) in dried blood spots (DBS) was quantified. C-reactive protein (CRP) was measured as an inflammatory marker.

**Results:**

At the 12-month visit, 59/80 (74%) in the intervention and 48/75 (64%) in the control arm achieved VS (the same proportion as at 6 months; *P* = .2); TFV-DP concentrations (median, IQR) in DBS were significantly higher in the intervention arm: 884 (491–1296) vs 598 (239–964) fmol/3-mm DBS punch in the control arm (*P* < .01). Higher TFV-DP DBS concentrations correlated with a slight decrease in CRP (Spearman's rho = −0.19; *P* = .02).

**Conclusions:**

UTRA-enhanced adherence support did not result in a significantly higher VS rate but was associated with increased TFV-DP in DBS—in turn, associated with lower CRP levels—suggesting that UTRA-enhanced adherence support improves long-term drug exposure and could also reduce HIV-associated inflammation.

**Clinical Trials Registration:**

clinicaltrials.gov (https://clinicaltrials.gov/study/NCT05333679).

## Teaser

In a randomized controlled trial, urine tenofovir rapid assay enhanced adherence-support improved long-term adherence, as measured by tenofovir-diphosphate in dried blood spots, but did not increase viral load suppression in the intervention arm compared to the control arm.

Achieving virologic suppression (VS) in 95% of patients receiving antiretroviral therapy (ART) is the third goal of the UNAIDS 95-95-95 targets. Adequate adherence is key to maintaining VS and preventing human immunodeficiency virus (HIV) drug resistance (HIVDR) for a sustainable public health ART program in resource-limited settings. People with HIV (PWH) who initiate ART with a fixed-dose combination of tenofovir, lamivudine, and dolutegravir (TLD), or who transition to TLD while having VS, have a very low risk of drug resistance, when subsequently presenting with virologic failure (VF). In contrast, approximately 20% of PWH who transitioned from a nonnucleoside reverse transcriptase inhibitor (NNRTI)–based regimen to TLD while viremic, and who subsequently experience VF on TLD, develop dolutegravir-associated drug resistance [1].

**Table 1.** Baseline Participant Characteristics

	Control (n = 100)	Intervention (n = 100)	*P*
Female sex, No. (%)	66 (66)	71 (71)	.5
Age, median (IQR), y	43 (34.75–51)	43 (35.75–51)	.8
CD4 count, median (IQR), cells/μL	347 (219–526.5)	308 (187–497)	.2
Baseline VL, median (IQR), copies/mL	19 (19–180)	33 (19–223)	.2
Months treated at screening, median (IQR)	11.4 (0.03–22.08)	10.5 (0.92–21.31)	.47
Months since last VL >50 copies/mL, median (IQR)	2.9 (0.39–4.70)	2.72 (0.85–4.48)	.8
Patients on TDF/3TC/DTG, No. (%)	72 (72)	74 (74)	.9

For continuous variables, data are summarized with medians and IQRs. The majority of participants received a regimen of TDF, 3TC, and DTG. *P* values were determined with Wilcoxon rank-sum tests.

Abbreviations: DTG, dolutegravir; HIV, human immunodeficiency virus; TDF, tenofovir disoproxil fumarate; VL, viral load; 3TC, lamivudine.

Apart from drug resistance, another consequence of inadequate adherence is increased inflammation. In the Strategic Timing of AntiRetroviral Treatment (START) study, incomplete adherence, as assessed by self-report, was associated with increased interleukin-6 (IL-6) levels even when participants had VS [2], and in the Multicenter AIDS Cohort Study, incomplete adherence was associated with higher levels of multiple inflammatory markers, including C-reactive protein (CRP) [3]. Moreover, lower adherence assessed by more objective metrics was associated with elevated CRP and other inflammatory markers, despite VS [4, 5]. Scalable strategies to detect inadequate adherence early to provide behavioral support to avoid ultimate VF and inflammation are therefore a high priority.

Accessible measures of determining adherence, such as self-report or visual analog scales, are limited by subjectivity and “white coat” bias; pharmacy refill adherence assessment is not sufficiently sensitive [6]. While electronic adherence monitoring and reminders provide a valuable alternative to self-report, affordability limits access to these technologies in large public health programs in low- and middle-income countries (LMICs) [7, 8].

The urine tenofovir (TFV) rapid assay (UTRA) is a qualitative competitive lateral flow assay to use at the point of care, which shows a positive test line when TFV is below the limit of detection. It provides an easy-to-use and acceptable low-cost strategy to assess adherence to fixed-dose combination ART regimens that include TFV [9]. Adherence to pre-exposure prophylaxis (PrEP), as measured with UTRA, predicted future PrEP discontinuation [10] and counseling with this test increased long-term adherence to PrEP in a randomized trial [11]. In a previous cross-sectional study of participants receiving TFV containing ART, we showed that urine TFV detected by UTRA in combination with VF predicted drug resistance in patients on an efavirenz-based regimen [12]. We also found that, in patients at high risk of VF who received a boosted protease inhibitor regimen or dolutegravir regimen, the majority of patients with VF had undetectable TFV in urine [13]. Although counseling with the UTRA increased VS in a study using a pre-post intervention design [14], the test has not yet been studied among PWH receiving ART in a randomized controlled trial (RCT).

We therefore conducted an RCT using UTRA-informed adherence support as the intervention, and assessed VS at 12 months as the primary outcome, with VS at 6 months as the secondary outcome. We also explored the impact of the intervention on drug exposure and inflammatory markers in patients randomized to UTRA-enhanced adherence support compared with the standard of care.

## METHODS

### Setting

The study site is a primary health care facility in Gugulethu, Cape Town, South Africa. Gugulethu (∼400 000 residents) is a high-density and predominantly lower socioeconomic status neighborhood. The primary healthcare facility where recruitment of participants and data collection took place is among the largest ART clinics in Cape Town, with more than 8500 patients enrolled in HIV care as of 2021 (Personal communication - study site manager).

### Study Design

We conducted a 2-arm, open-label RCT (https://clinicaltrials.gov/study/NCT05333679). Adult PWH (≥18 years) were enrolled from a public sector health program where routine HIV-1 RNA load (VL) testing is performed between 4 and 6 months, at 12 months, and thereafter annually per guidelines [15]. People with HIV were eligible if they were taking or initiating an antiretroviral regimen containing at least 1 drug with a high genetic barrier to resistance (eg, dolutegravir, atazanavir/ritonavir, darunavir/ritonavir or lopinavir/ritonavir and tenofovir disoproxil fumarate [TDF]) and if they had experienced a raised VL (≥50 copies/mL) at any time after ART initiation (Table 1). Eligible participants were randomly assigned, through electronic random-number generation by the data manger sent in a closed envelope delivered to the study site, in a 1:1 ratio to the UTRA intervention compared with standard of care after signing written informed consent. Laboratory personnel were blinded to the assignment group.

**Figure 1.**
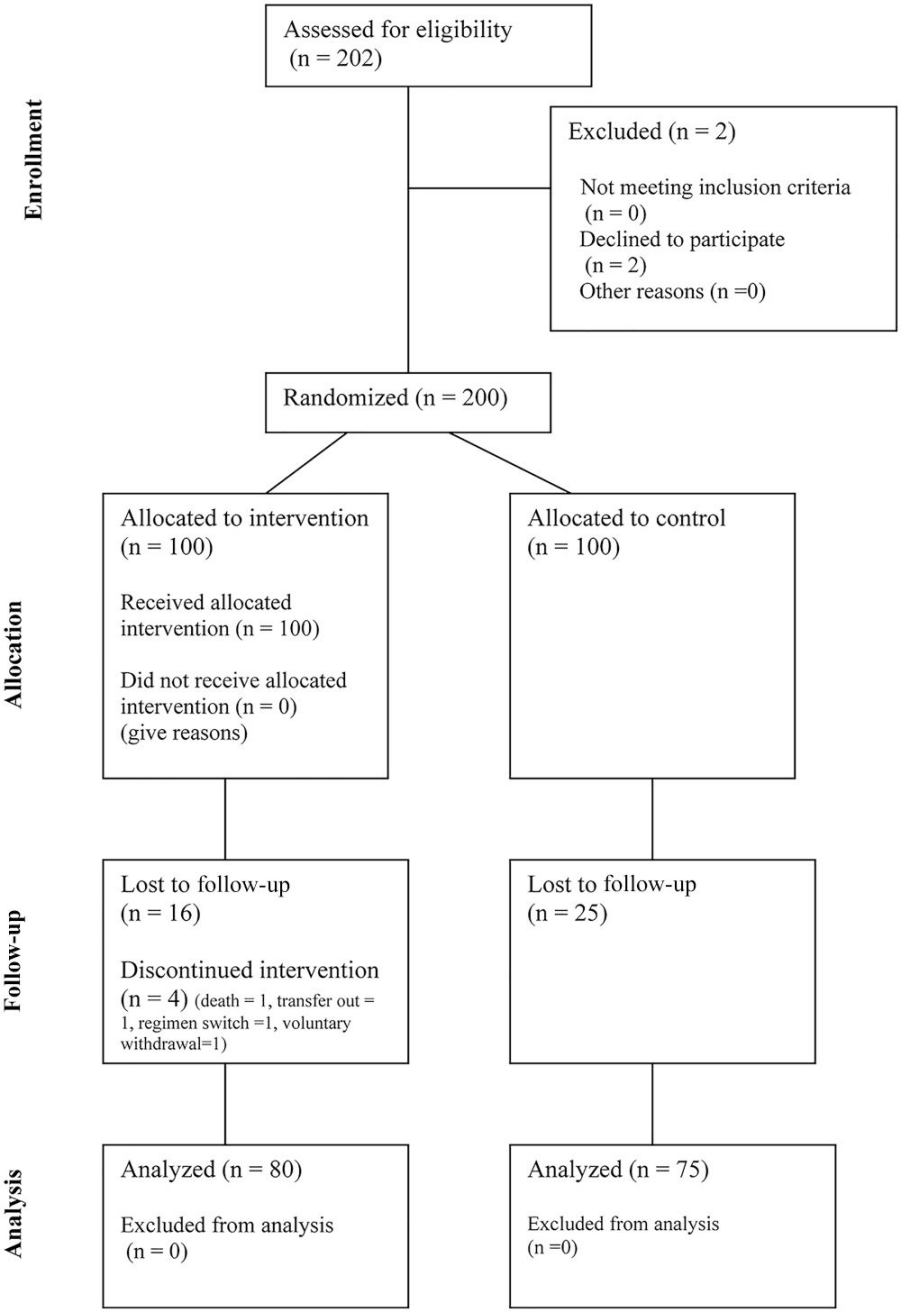
CONSORT (Consolidated Standards of Reporting Trials) diagram of the study.

All participants who were enrolled continued to receive ART and care (including annual VL testing) from their local public sector clinic. Study visits were every 3 months up to month 12. At each visit all participants had their ART regimen confirmed, and adherence to ART was checked using self-report and pharmacy refill measures. In addition, participants in the intervention arm were offered a UTRA with real-time results, followed by immediate data-driven adherence counseling by skilled study counsellors explaining the importance of the UTRA result. Participants with undetected urine TFV or a raised VL in the UTRA intervention arm were referred to the clinic for standard-of-care adherence support, with undetectable urine TFV managed similarly to a current VL of more than 50 copies/mL. Participants in the control arm received standard-of-care adherence support as per the South African National Department of Health guidelines [16]. The standard-of-care adherence support services for participants are triggered by a current VL greater than 50 copies/mL and included a counseling session with a clinician and referral to peer counseling sessions involving a client-focused strategy—assessing the barriers to adherence and discussing effective strategies aligned to the cause of nonadherence and that are acceptable to the clients. Every 6 months (baseline, month 6 and 12), blood samples were drawn for VL testing and stored for retrospective HIV drug resistance testing, plasma TFV concentrations, TFV diphosphate (TFV-DP) concentrations in dried blood spots (DBS), as well as for the inflammatory biomarker CRP, for the predefined exploratory outcomes.

The primary outcome was the proportion of participants in each arm achieving VS to less than 50 copies/mL compared with the alternative, viral breakthrough (VB) by month 12. Secondary outcomes included retention in care at month 12, VS (<50 copies/mL) at month 6, and feasibility and acceptability measured at enrollment and at months 6 and 12, with standardized questionnaires. In this article we report on the primary and secondary virologic outcomes and explore the relationship of the intervention with drug exposure and inflammation (CRP). Qualitative outcomes will be reported separately.

### Ethics

The study was approved by University of Cape Town Health Research Ethics Committee (HREC) and Stellenbosch University HREC (reference: 587/2021). All enrolled participants provided written informed consent and were compensated according to institutional guidelines.

### Diagnostic Investigations

Qualitative urine TFV testing in the intervention arm was performed with the UTRA, with an adherence window of 24–48 hours, as previously described [12, 13, 17]; DBS and plasma for TFV concentrations and VL were collected at each study visit. Dried blood spots were stored at −20°C and plasma stored at −80°C until analysis. Viral load testing was performed with the Abbott Alinity m HIV-1 assay (Abbott Park, IL, USA), which has a lower limit of detection of 20 copies/mL, performed retrospectively on stored samples collected at study visits.

#### Pharmacologic Assays

Tenofovir diphosphate (with an adherence window of 6–8 weeks) was analyzed using liquid chromatography/tandem mass spectrometry (LC-MS/MS) with a validated method previously described, with a quantification range of 27–6924 fmol/3-mm DBS punch [18]. Plasma TFV was quantified by LC-MS/MS using a validated method with a quantification range of 10–2000 ng/mL [19] (Supplementary Material 1).

#### Drug Resistance Testing

In samples with a VL more than 400 copies/mL, here defined as having VF, HIV drug resistance testing was performed with a previously described method and bioinformatics pipeline [20, 21] and interpreted with the Stanford HIV database version 9.6 (Supplementary Material 2).

### Statistical Analysis

#### Sample Size Calculation

Based on expecting 80% suppression (VL <50 copies/mL) in the control group, a sample size of 200 (intervention and control) would enable the detection of an increase in VS to 93% with 80% power (2-sample proportion test).

Participants missing the 12-month visit were excluded from per-protocol (PP) analysis but included in intention-to-treat (ITT) analysis as non-suppressed.

For the determination of sensitivity and specificity, a positive urine test was defined as absent urine TFV and a “positive outcome” as VB. All statistical analyses were performed in R (R Foundation for Statistical Computing, Vienna, Austria) [22]. Continuous outcomes, by group, were assessed with the Wilcoxon rank-sum test and categorical outcomes with Pearson's chi-square test. A *P* value of .05 was regarded as significant. Optimal quantitative assay cutoffs were determined with receiver operator characteristics (ROC).

## RESULTS

### Participants

Two hundred participants with a history of VB were enrolled between 2 March 2022 and 25 August 2023. Enrollment and retention are shown in the CONSORT (Consolidated Standards of Reporting Trials) diagram (Figure 1).

### Diagnostic Findings

#### Viral Load Outcomes

In the PP analysis, of the 155 month-12 visits, 107 had VS: 59 of 80 (74%) in the intervention arm and 48 of 75 (64%) in the control arm (*P* = .2). Using an ITT analysis, 59 of 100 in the intervention arm compared with 48 of 100 in the control arm achieved VS (*P* = .12). At the 6-month time point, 49 of 66 (74%) in the intervention compared with 43 of 67 (64%) in the control arm (*P* = .2) had VS. These differences were not statistically significant.

#### Drug Concentrations

**Figure 2.**
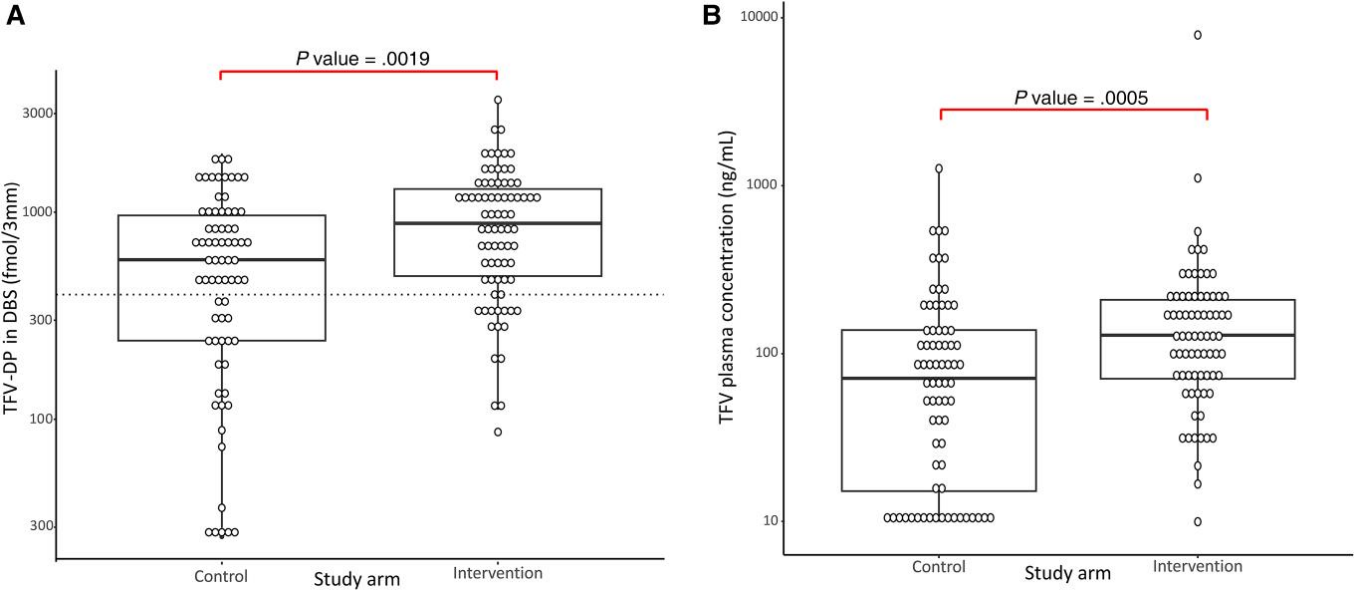
*A*, Tenofovir-diphosphate (TFV-DP) in dried blood spots (DBS) by study arm. The horizontal dashed line indicates the threshold (400 fmol/3-mm DBS punch) that is predictive of virologic failure in a prior study among African patients [23]. Groups were compared with Wilcoxon rank-sum tests. *B,* Tenofovir (TFV) plasma concentrations by study arm. Groups were compared with Wilcoxon rank-sum tests.

Tenofovir drug exposure was significantly higher in the intervention arm at month 12. The median (IQR) TFV-DP concentrations in DBS in the intervention arm were 884 (491–1296) compared with 598 (239–964) fmol/3-mm DBS punch (*P* < .01) in the control arm, and median (IQR) TFV plasma concentrations in the intervention arm were 129 (70.9–209) compared with 71 (15.2–138) ng/mL in the control arm (*P* < .01) (Figure 2*A* and 2*B*).

We further explored the imperfect correlation between improved short-term and long-term drug exposure and VL outcome by combining data from both arms. The TFV-DP concentration in DBS at month 12 was significantly higher in participants from both arms with VS with a median (IQR) of 838 (499–1291) compared with 372 (133–844) fmol/3-mm DBS punch in those with VB (*P* < .01). No cutoff provided a good prediction of VB; the cutoff that maximized the sum of sensitivity and specificity was 332 pmol/3-mm DBS punch (sensitivity of 47% and specificity of 90% for VB; area under the ROC curve [AUC]: 0.74). The TFV plasma concentrations were not significantly higher in participants with VS (median: 99.6 ng/mL; IQR: 57.9–198 ng/mL) compared with those with VB (median: 90.3 ng/mL; IQR: 15.2–183 ng/mL) (*P* = .13). No cutoff provided a good prediction of VB (AUC: 0.58).

### Sensitivity of Urine TFV Testing to Predict VB at Baseline

At the baseline visit, 11 of 42 with VB had a positive UTRA, with a sensitivity of 26% for concurrent VB, whereas of 58 with VS, 53 had detectable TFV in urine, providing a specificity of 91%.

### Urine TFV Assay Persistence

Of the 80 participants in the intervention arm retained at 12 months, 21 had a VL of 50 copies/mL or greater. Of these, only 6 had undetectable TFV in urine in any preceding visit (at months 0, 3, 6, and 9), giving a sensitivity of 28.6% for predicting viremia at 12 months. Of the 59 with suppressed VLs at 12 months, 47 had persistently detectable urine TFV, providing a specificity of 79%.

### Inflammatory Biomarkers

**Figure 3.**
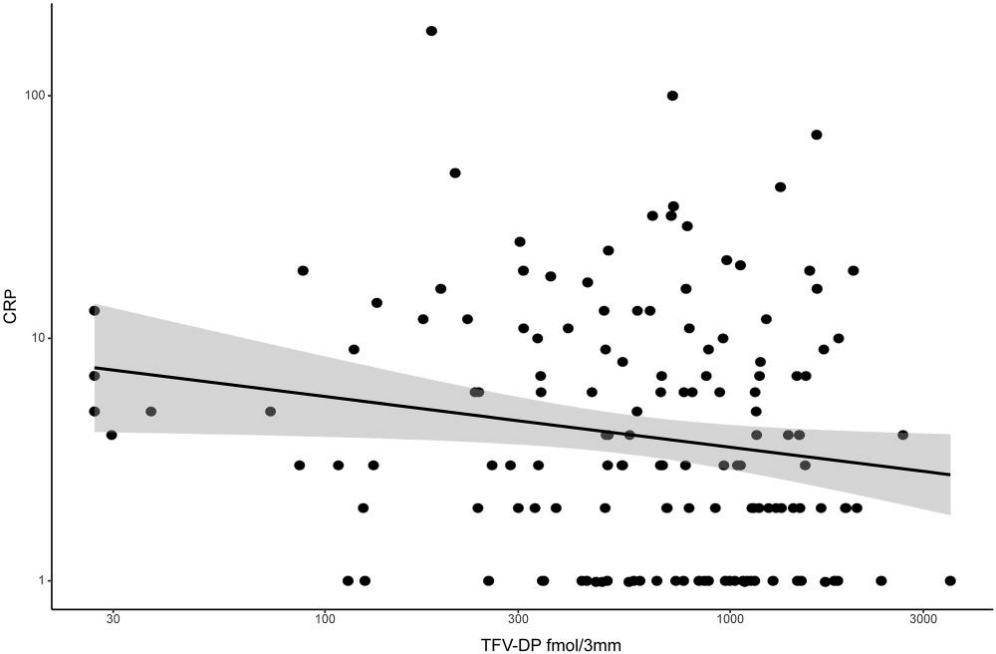
Decreased C-reactive protein (CRP) inflammatory marker with increasing tenofovir-diphosphate (TFV-DP) in dried blood spot (DBS) concentrations.

At month 12, the median (IQR) CRP in the intervention arm was 2.5 mg/L (1–7 mg/L) compared with 5 mg/L (1–9 mg/L) in the control arm (*P* = .056), which did not meet the significance threshold. When combining data across the arms, CRP levels decreased slightly but significantly with increasing concentrations of TFV-DP in DBS (Figure 3).

### HIV Drug Resistance

Of the 48 individuals with VB at month 12, 23 (48%) had an VL greater than 400 copies/mL, the predefined threshold for drug resistance sequencing; all had successful drug resistance sequencing (Supplementary Material 3 provides GenBank accession numbers). Overall, HIV drug resistance did not provide an explanation for VF: only 1 participant of 6 on a protease inhibitor (PI)–based regimen had major PI mutations in addition to nucleoside reverse transcriptase inhibitor (NRTI) mutations. Of the 16 participants on a DTG regimen, none had major integrase strand transfer inhibitor (INSTI) mutations, with 4 having a single NRTI mutation and 1 of these having a single major PI mutation (Supplementary Material 4).

## DISCUSSION

We conducted an RCT among PWH with adherence challenges using the UTRA for the first time. Adherence counseling was informed by UTRA in the intervention arm compared with standard-of-care adherence counseling in the control arm. However, participants in the intervention arm did not have a significantly higher rate of VS than controls. Nevertheless, TFV-DP concentrations in DBS, indicative of adherence over the prior 6–8 weeks, and TFV plasma concentrations, a short-term adherence indicator (∼3–5 days before the visit), were higher among the intervention-arm participants than the control arm. The improved long-term adherence assessed with TFV-DP correlated with a slight, but significant reduction in CRP, a systemic inflammatory marker.

There are several likely reasons for the primary outcome null finding. The sensitivity of the UTRA of only 26% in detecting concurrent VL greater than 50 copies/mL at baseline and 29% for predicting future virologic failure suggests that the UTRA as a stand-alone test has limited utility in identifying patients with poor adherence when used in a similar context to this study. Any recent medication adherence improvement before the UTRA visit, due to social desirability bias, could result in detectable urine TFV, as 14% of patients who took their last dose 4 days before would still have detectable urine TFV due to the assay's high analytical sensitivity [24]. Our original hypothesis had been that UTRA would improve adherence by detecting patients with poor adherence, enabling the reinforcement of adherence. Nevertheless, UTRA improved adherence, despite the low sensitivity to detect VB. This may be explained by an overall positive impact on care in providing another opportunity for an adherence discussion and positive reinforcement. These aspects of the UTRA are currently being investigated as part of a qualitative aim of the study.

Another reason for the null finding is that a single VL measure serves as an indirect measure of adherence in both study arms. Highly robust regimens such as TLD are “forgiving” of missed doses. As a result, VB may not occur or be detected at infrequent VL testing visits despite relatively low levels of adherence [25]. A relative low correlation between VL and drug exposure diluted the effect size of the intervention on the primary VL outcome. This effect size was smaller than our study assumptions. Based on the measured proportion of VS in the intervention and control arms, a much larger sample size (total N ≥ 350, with equal numbers in the intervention and control arms) would be required to reach statistical significance in the difference of VS rates (Pearson's chi-square *P* < .05).

Although drug exposure was an exploratory outcome, it is nevertheless important as low adherence between visits can result in both inflammation and subsequent cardiovascular risk, as evidenced in our study and others, and potentially in the loss of VS between infrequent VL testing visits [4, 5]. An improvement in drug exposure is therefore an important outcome of this study [3].

We did not detect any individuals with INSTI mutations, but only 23 had an VL greater than 400 copies/mL, and the expected prevalence is low [1]. Moreover, due to the fitness cost of major INSTI mutations [26], relative high levels of adherence may be required for resistance selection, whereas VF in this study is largely attributable to low drug exposure.

Our study, albeit small, differed from existing studies in that it was the first RCT of urine TFV assay-informed counseling compared with standard-of-care counseling in improving ART outcomes. A larger RCT is in progress to detect the effects of the UTRA on VS. Urine TFV testing has been shown to improve adherence in patients receiving PrEP with TDF/emtricitabine [11] and these assays could also detect TFV in participants using tenofovir alafenamide (TAF) [27, 28]. In another study of participants receiving efavirenz-based ART in Uganda and South Africa, the prevalence of HIV drug resistance was 204 of 224 (91%) among participants with detected urine TFV compared with 39 of 59 (66%) in those without detected urine TFV [29]. In a nested case-control study within the ADVANCE RCT, 30.7% (70/228) with VL of 200 copies/mL or greater had detectable urine TFV compared with 100% (53/53) of controls with HIV VL less than 50 copies/mL, whereas NRTI drug resistance was detected in 50% (10/20) of cases with detectable urine TFV compared with 8.3% (2/24) of cases with undetectable urine TFV concentrations [30]. Studies to assess the utility of UTRA combined with VL testing as a prescreening tool for HIV drug resistance testing are ongoing and should shed light on its utility in algorithms to select individuals who require HIV drug resistance testing [31]. Finally, a recently published study showed that the UTRA increased VS among PWH using a pre-post intervention design [14].

This study had several limitations, which could have increased bias and random error. The study was from a single site, the sample size relatively small, and the observed effect size of the intervention on VS was smaller than assumed. We were thus not able to show whether the intervention was able to improve rates of VS. Although TFV-DP analysis was planned, this was explorative and not a primary outcome. The investigation of inflammatory markers was a planned exploratory investigation, with CRP chosen based on its association with adherence [3]. Although CRP was weakly negatively correlated with drug exposure, we did not show a difference in CRP between study arms and were not able to show its long-term clinical impact. Conclusive evidence would require reproduction in other settings or a larger sample size.

In conclusion, a recently developed urine TFV assay, which is expected to be low cost and is easy to administer, did not result in a significantly higher rate of VS in the intervention arm when used in an enhanced adherence intervention. However, the urine assay improved both short- and long-term ART exposure. Improved adherence also resulted in lower levels of CRP as a marker of inflammation. This assay may therefore have utility as an accessible adherence-support tool for LMICs. Additional and larger studies are needed to confirm the possible placement of UTRA in ART programs, in achieving VS, and in algorithms in combination with concurrent VL testing to triage drug resistance testing among PWH whose virologic failure is unexplained by poor adherence [31].

## Supplementary Material

ciaf337_Supplementary_Data

## Supplementary Data

Supplementary materials are available at *Clinical Infectious Diseases* online. Consisting of data provided by the authors to benefit the reader, the posted materials are not copyedited and are the sole responsibility of the authors, so questions or comments should be addressed to the corresponding author.

## Notes

***Author contributions.*** G. U. v. Z. wrote the first draft of the manuscript, which was reviewed by M. G., E. D., C. O., L. J., M. v. S., T. K., and E. D. oversaw the TFV-DP in DBS analysis and G. U. v. Z. oversaw the drug resistance testing. All authors reviewed and approved the final manuscript.

***Acknowledgments.*** The authors acknowledge Tshegofatso Mahlangu, Carli Gordijn, and Nicola Coetzee for sample processing and Charne Dixon for pharmacological assays.

***Data availability.*** Accession numbers for sequences are provided in supplement 3. Any other deidentified data can be requested from the study investigators.

***Financial support.*** Research reported in this article was supported by the US National Institutes of Health (NIH) and South African Medical Research Council through its US-SA Program for Collaborative Biomedical Research (grant number R01AI152119). Urine assays were provided by National Institute of Allergy and Infectious Diseases (NIAID)/NIH grant number 2R01AI143340 (Principal Investigator M. G.).
