# Supplementary material for: A Real-Time Urine Tenofovir Assay Improves Drug Adherence Among People With HIV With Prior Virologic Failure in a Randomized Controlled Trial
Source: Clin Infect Dis. 2025 Jun 20;81(5):e352–9. doi: 10.1093/cid/ciaf337 (PMC12728291; doi:10.1093/cid/ciaf337)
Supplement: ciaf337_Supplementary_Data [file ciaf337_supplementary_data.zip › Supp 3 Accession numbers.docx]

| **Sequence ID** | **GenBank accession number** |
| --- | --- |
| UTRA-3 | PV275329 |
| UTRA-4 | PV275330 |
| UTRA-7 | PV275333 |
| UTRA-11 | PV275334 |
| UTRA-14 | PV275337 |
| UTRA-16 | PV275339 |
| UTRA-21 | PV275342 |
| UTRA-23 | PV275343 |
| UTRA-25 | PV275345 |
| UTRA-35 | PV275296 |
| UTRA-37 | PV275298 |
| UTRA-40 | PV275301 |
| UTRA-47 | PV275289 |
| UTRA-48 | PV275307 |
| UTRA-51 | PV275309 |
| UTRA-55 | PV275312 |
| UTRA-56 | PV275313 |
| UTRA-66 | PV275288 |
| UTRA-59 | PV275316 |
| UTRA-61 | PV275318 |
| UTRA-63 | PV275320 |
| UTRA-64 | PV275321 |
| UTRA-72 | PV275327 |
