# Supplementary material for: A Real-Time Urine Tenofovir Assay Improves Drug Adherence Among People With HIV With Prior Virologic Failure in a Randomized Controlled Trial
Source: Clin Infect Dis. 2025 Jun 20;81(5):e352–9. doi: 10.1093/cid/ciaf337 (PMC12728291; doi:10.1093/cid/ciaf337)
Supplement: ciaf337_Supplementary_Data [file ciaf337_supplementary_data.zip › Supplement 1_pharmacologic methods.docx]

**Supplement: Detailed pharmacological methods (Tenofovir diphosphate in dried blood spots and Tenofovir in plasma quantification).**

**Materials and methods**

Tenofovir diphosphate reference standard was obtained from Moravek Inc. (Brea, USA). Tenofovir and tenofovir-d6 (internal standard, ISTD) were obtained from LGC Toronto Research Chemicals (Vaughan, Canada). Analytical grade Potassium chloride, ammonium acetate and dichloromethane were obtained from Labchem (Edenvale, South Africa). Strata-X 33u Polymeric Reversed Phase 200mg/3mL solid phase extraction (SPE) cartridges were obtained from Phenomenex (Torrance, USA) and Sep-Pak Accell Plus QMA 3 cc Vac Cartridge, 500 mg Sorbent per Cartridge, 37 - 55 μm from Waters (Milford, USA). Lyophilized potato acid phosphatase (~2 units/mg protein) was obtained from Roche (Basel, Switzerland). LCMS grade Formic acid was obtained from Fisher Scientific (Massachusetts, United States). Analytical grade Millipore water was filtered in-house using a Synergy UV Water Purification System (Ultrapure Type 1 water) with a Biopak Polisher from Merck Millipore (Darmstadt, Germany). Methanol and acetonitrile for HPLC use were obtained from ROMIL Pure Chemistry (Cambridge, United Kingdom). LC-MS grade ammonium formate and Whatman 903 protein saver cards were obtained from Sigma-Aldrich/Merck (St Louis, USA).

**Instruments**

Analysis was performed on a Shimadzu LCMS-8040 triple quadrupole mass spectrometer connected to a Shimadzu Prominence liquid chromatography system (Kyoto, Japan). This included solvent delivery pumps (LC-20AD XR), an autosampler (Nexera SIL-20AC XR), and a temperature-controlled column compartment (CTO-20A). Data acquisition and processing were carried out using LabSolutions software (version 5.109). Separation was achieved on a Poroshell 120EC-C18 column (2.7 μm, 3.0 x 100 mm) column manufactured by Agilent Technologies (California, United States). A SPE Ware CEREX System 48-II Positive Pressure Processor (California, United States) was utilized for solid phase extraction, and a Stuart sample Concentrator and Block Heater was used to concentrate extracted samples (Staffordshire, United Kingdom).

**Chromatographic method**

For the quantitation of tenofovir, mobile phase A and B consisted of 2 mM ammonium formate and 0.1% formic acid in water, and methanol, respectively. A flow rate of 0.45 mL/min was used with an isocratic flow of 70:30 (v/v, A:B) with a 5 min analysis time. For both the plasma and DBS methods, a quadratic regression with 1/c (where c is concentration) weighting was utilized for quantification. Carry-over was mitigated with a needle wash of methanol:water (1:1, v/v) before and after aspiration. Column temperature was maintained at 30֯C and autosampler temperature at 15֯C.

**Preparation of calibration standards and quality controls**

For tenofovir quantitation in plasma, working solutions were prepared in water from a 1 mg/mL stock solution of tenofovir to achieve concentrations of 100, 50.0, 25.0, 11.2, 5.00, 2.50, 1.00 and 0.500 µg/mL. A volume of 40.0 µL of each stock solution was spiked into 1.96 mL of K2EDTA plasma, to yield calibrator concentrations of 2000, 1000, 500, 225, 100, 50, 20.0 and 10ng/mL. Similarly, 40 µL of working solutions at concentrations of 80.0, 40.0, 1.25 and 0.500 µg/mL were used to spike 1.96 mL of K2EDTA plasma to achieve QC concentrations of 1600 (QC high), 800 (QC medium), 25.0 (QC low) and 10.0 ng/mL (LLOQ).

For the preparation of tenofovir-diphosphate standards and QC’s, mass was used to adapt the method from a single 3 mm spot to a whole DBS (representing an accurate 50 µL blood volume). Working solutions of tenofovir-diphosphate were prepared in water to yield concentrations of 3.91, 7.81, 15.6, 31.3, 62.5, 125, 250, 500 and 1000 ng/mL. After the indirect measurement of tenofovir diphosphate, the concentration of tenofovir-diphosphate in the calibration standards was calculated to be 27.0, 54.1, 108, 216, 433, 866, 1731, 3462 and 6924 fmol/3 mm spot. For the quality control working solutions, tenofovir-diphosphate concentrations of 3.91 (LLOQ), 10.0 (QC low), 400 (QC medium) and 800 ng/mL (QC high) were prepared in water, to yield final QC concentrations of 27.1 (LLOQ), 69.2 (QC low), 2770 (QC medium) and 5539 (QC high) fmol/3 mm punch.

**Extraction Procedure**

For tenofovir quantitation in plasma, all calibrators, quality controls, blanks and patient samples were thawed at room temperature and a 50 µL volume was transferred to a 1.5 mL microcentrifuge tube for extraction. A volume of 300 µL of cold ACN: methanol (1:1, v/v) containing tenofovir-d6 as ISTD at 100 ng/mL was added to each sample, except the double blank to which ISTD-free solvent was added. The sample was vortexed for 40 s, and centrifuged at 17000 x g for 6 min at room temperature. A volume of 250 µL of the supernatant was transferred to a borosilicate tube, and the sample was dried down under a gentle flow of nitrogen at 30֯C. Samples were reconstituted with 200 µL of water, vortexed at high speed for 40 s, and the sample was transferred to a clean microcentrifuge tube. These were centrifuged at 17000 x g for 6 min at room temperature, and transferred to a 96-well plate. A volume of 10 µL was injected onto the LC-MS.

For the extraction of tenofovir diphosphate from dried blood spots, the method described by Bushman et al (2011) was used, with the following adaptations. Due to the use of a less sensitive LC-MS instrument, a whole dried blood spot (as opposed to a 3 mm punch), prepared from an accurate 50 µL volume of blood on Whatman 903 protein saver cards, was extracted for analysis. Lysate was prepared by adding 1 mL of Methanol:water (70:30, v/v) to each whole DBS, followed by sonication on high power for 10 min and vortexing at high speed for 1 min. The lysate was centrifuged at 16000 x g for 5 min at room temperature.

Dephosphorylation was performed on QMA cartridges which were conditioned with 2 mL of water and equilibrated with 1.5 mL of 1 M KCl, and 2 mL of 5 mM KCl. A volume of 0.5 mL of lysate was added to the cartridge, together with 25 µL of the respective calibrator or QC working solutions, or 25 µL of water for the blanks and patient samples. Elutions were performed with 5 mL of 75 mM KCl and 7 mL of 95 mM KCl. Finally, the elution using 2 mL of 1 M KCl was collected into borosilicate glass tubes. The potato acid phosphatase enzyme concentration was optimized for the use of a whole DBS and was prepared at 18 mg/mL in 1 M sodium acetate, and 100 µL was added to each sample. The samples were vortexed for 20 s, and incubated in a heating block at 37֯C for 1 hour. Thereafter, the samples were cooled at 4֯C for 15 min, and 25 µL of tenofovir-d6 (ISTD) at 500 ng/mL was added to each sample. The samples were desalted on STRATA-X columns which were conditioned with 2 mL methanol and equilibrated with 4 mL of 15 mM ammonium acetate. The samples were added to the columns, followed by elution with 2 mL of 10 mM ammonium acetate and 0.5 mL of dichloromethane. The cartridges were thoroughly dried, prior to elution with 1 mL of methanol, which was eluted into borosilicate glass tubes and dried under a gentle stream of nitrogen at 40֯C. The samples were reconstituted in 150 µL of water, vortexed for 30 s and transferred to a 96-well plate. A volume of 30 µL was injected onto the LC-MS method described above for the quantification of tenofovir. The on-instrument calibration range was 3.91 to 1000 ng/mL for tenofovir, to which a conversion factor was applied to obtain a unit equivalent of tenofovir-diphosphate in fmol/3 mm punch, as per the original publication by Bushman et al (2011).

**References:**

Bushman LR, Kiser JJ, Rower JE, Klein B, Zheng J-H, Ray ML, Anderson PL. Determination of nucleoside analog mono-, di-, and tri-phosphates in cellular matrix by solid phase extraction and ultra-sensitive LC–MS/MS detection. Journal of Pharmaceutical and Biomedical Analysis, Volume 56, Issue 2, 2011, Pages 390-401, <https://doi.org/10.1016/j.jpba.2011.05.039>
