# Supplementary material for: A Real-Time Urine Tenofovir Assay Improves Drug Adherence Among People With HIV With Prior Virologic Failure in a Randomized Controlled Trial
Source: Clin Infect Dis. 2025 Jun 20;81(5):e352–9. doi: 10.1093/cid/ciaf337 (PMC12728291; doi:10.1093/cid/ciaf337)
Supplement: ciaf337_Supplementary_Data [file ciaf337_supplementary_data.zip › Supplement 2_drug resistance assay methods.docx]

**Supplement: Detailed methods of HIV drug resistance assay.**

**ONT pol drug resistance assay**

1.1 Reverse Transcription and PCR Amplification of HIV-1 pol

Nucleic acid was extracted from 500 µL of plasma using the NucliSENS easyMAG automated system (bioMérieux, France) following the manufacturer’s protocol.

Complementary DNA (cDNA) synthesis was done with either a one-step or two-step RT-PCR approach with the SuperScript IV First-Strand Synthesis System (Thermo Fisher Scientific, USA) and specific reverse primers R1a and R1b. PCR amplification was carried out using the Platinum SuperFi II PCR Master Mix (Thermo Fisher Scientific, USA).

A nested PCR targeting the HIV-1 *pol* gene—covering the protease (PR), reverse transcriptase (RT) and integrase (IN) regions—was performed with the primers listed in Table 1[1]. The thermocycling conditions are detailed in Table 2 using touchdown PCR approach to enhance specificity.

Table 1: Amplification primers for the HIV-1 *pol* gene

| Primer Name (direction) | Position^a^ | Sequence 5’ – 3’ |
| --- | --- | --- |
| Primers used in RT-PCR/pre-nested PCR | | |
| F1a (forward) | 1488 – 1505 | GGG AAG TGA YAT AGC WGG AAC |
| F1b (forward) | 1493 – 1511 | GTG AYA TAG CWG GAA CTA CTA G |
| R1a (reverse) | 5195 – 5213 | TAG TGG GAT GTG TAC TTC TGA AC |
| R1b (reverse) | 5197 – 5214 | TAG TGG GAT GTG TAC TTC TGA |
| Primers used in nested PCR | | |
| F2 (forward) | 2041 – 2066 | GGA AAG GAA GGA CAC CAA ATG AAA GAY TG |
| R2 (reverse) | 5054 – 5072 | TGC CAC ACA ATC AKC ACC TGC C |
| ^a^ According to HXB2 reference sequence (GenBank accession no K03455) | | |

Table 2: Thermocycling conditions of the 3kb *pol* assay

| **Touchdown RT-PCR thermal cycling conditions** | | | |
| --- | --- | --- | --- |
| Step | Temperature (°C) | Time (min) | Number of cycles |
| Reverse transcription | 50 | 50:00 | 1 X |
| RT inactivation/initial denaturation | 98 | 2:00 | 1 X |
| Amplification (Phase 1) | 98 | 0:10 | 5 X |
|  | 58 | 0:15 |  |
|  | 72 | 3:00 |  |
| Amplification (Phase 2) | 98 | 0:10 | 15 X |
|  | 58 – 50^a^ | 0:15 |  |
|  | 72 | 3:00 |  |
| Amplification (Phase 3) | 98 | 0:10 | 15 X |
|  | 50 | 0:15 |  |
|  | 72 | 3:00 |  |
| Final extension | 72 | 6:00 | 1 X |
| Reaction stop | 4 | Indefinitely | Hold |
| **Nested touchdown PCR thermal cycling conditions** | | | |
| Step | Temperature (°C) | Time (min) | Number of cycles |
| Initial denaturation | 98 | 2:00 | 1 X |
| Amplification (Phase 1) | 98 | 0:10 | 5 X |
|  | 58 | 0:15 |  |
|  | 72 | 3:00 |  |
| Amplification (Phase 2) | 98 | 0:10 | 15 X |
|  | 58 – 50^a^ | 0:15 |  |
|  | 72 | 3:00 |  |
| Amplification (Phase 3) | 98 | 0:10 | 15 X |
|  | 50 | 0:15 |  |
|  | 72 | 3:00 |  |
| Final extension | 72 | 6:00 | 1 X |
| Reaction stop | 4 | Indefinitely | Hold |
| ^a^ ΔT = Decrease of 0.5°C per cycle | | | |

Amplification of the ~3 kb nested PCR products was verified by agarose gel electrophoresis. For samples that failed amplification, a two-fragment approach done to separately amplify the PR-RT and IN regions. For this, 1µL of the RT-PCR or pre-nested product, was used as input in separate second-round PCR reactions targeting PR-RT and IN, respectively. Amplification was performed using Platinum SuperFi II PCR Master Mix, with the primers listed in Table 3 and cycling conditions described in Tabel 4.

Table 3: Primers for alternative PR-RT and IN fragment amplification

| Primers used in two-fragment nested PCR | | | |
| --- | --- | --- | --- |
| Target | Primer Name (direction) | Position^a^ | Sequence 5’ – 3’ |
| PR-RT | F2 (forward) | 2041 – 2066 | GGA AAG GAA GGA CAC CAA ATG AAA GAY TG |
|  | RT_PR_short inner (reverse) | 4033 – 4065 | TGA TTC CTA ATG CAT ACT GTG AGT CTG TTA CWA |
| IN | Int_short inner (forward) | 4158 – 4186 | CCA GCA CAT AAA GGA ATT GGA GGA AAT GA |
|  | R2 (reverse) | 5054 – 5072 | TGC CAC ACA ATC AKC ACC TGC C |
| ^a^ According to HXB2 reference sequence (GenBank accession no K03455) | | | |

Tabel 4: Thermocycling conditions for the two-fragment PR-RT and IN amplification

| PR-RT fragment secondary touchdown PCR | | | | | | |
| --- | --- | --- | --- | --- | --- | --- |
| Step | Temperature (°C) | | Time (min) | | Number of cycles | |
| Initial denaturation | 98 | | 1:00 | | 1 X | |
| Amplification (Phase 1) | 98 | | 0:10 | | 5 X | |
|  | 58 | | 0:15 | |  |  |
|  | 72 | | 1:30 | |  |  |
| Amplification (Phase 2) | 98 | | 0:10 | | 15 X | |
|  | 58 – 50^a^ | | 0:15 | |  |  |
|  | 72 | | 1:30 | |  |  |
| Amplification (Phase 3) | 98 | | 0:10 | | 15 X | |
|  | 50 | | 0:15 | |  |  |
|  | 72 | | 1:30 | |  |  |
| Final extension | 72 | | 5:00 | | 1 X | |
| Reaction stop | 4 | | Indefinitely | | Hold | |
| IN fragment secondary touchdown PCR | | | | | | |
| Step | | Temperature (°C) | | Time (min) | | Number of cycles |
| Initial denaturation | | 98 | | 1:00 | | 1 X |
| Amplification (Phase 1) | | 98 | | 0:10 | | 5 X |
|  | | 58 | | 0:15 | |  |
|  | | 72 | | 1:00 | |  |
| Amplification (Phase 2) | | 98 | | 0:10 | | 15 X |
|  | | 58 – 50^a^ | | 0:15 | |  |
|  | | 72 | | 1:00 | |  |
| Amplification (Phase 3) | | 98 | | 0:10 | | 15 X |
|  | | 50 | | 0:15 | |  |
|  | | 72 | | 1:00 | |  |
| Final extension | | 72 | | 5:00 | | 1 X |
| Reaction stop | | 4 | | Indefinitely | | Hold |
| ^a^ ΔT = Decrease of 0.5°C per cycle | | | | | | |

1.2 Library Preparation, Nanopore Sequencing, and Data Analysis

For each successfully amplified sample, PCR products were purified using Agencourt AMPure XP beads (Beckman Coulter, USA) according to the manufacturer’s instructions. DNA concentrations were quantified using the Qubit dsDNA HS Kit and Qubit 2.0 Fluorometer (ThermoFisher Scientific, USA).

Library preparation was performed on the purified amplicons using the Nanopore Rapid Barcoding Kit 24 V14, SQK-RBK114.24 (Oxford Nanopore Technologies, UK), following the ONT protocol *Rapid Sequencing V14 – Amplicon Sequencing* Version RAA_9198_v114_revD_29Nov2023.

Sequencing libraries were loaded onto R10.4.1 Flongle flow cells (FLO-FLG114, Oxford Nanopore Technologies, UK) and sequencing was performed on a GridION for up to 24 h. High-accuracy basecalling was used in the MinKnow software with Guppy (version 7.1.4).

The resulting FASTQ files were processed using the Nano-RECall pipeline to generate consensus sequences[2]. The sequences were uploaded to the Stanford HIV Drug Resistance Database (Stanford HIVdb) for drug resistance interpretation.

References:

1. Coetzee N, Woods CK, Delaney K, et al. Full Pol-Gene PCR and Rapid ONT Library Preparation for Accurate Drug Resistance Sequencing. In: Conference on Retroviruses and Opportunistic Infections: CROI 2024, March 3-6. Denver, Colorado, USA: 2024.

2. Delaney KE, Ngobeni T, Woods CK, et al. Nano-RECall provides an integrated pipeline for HIV-1 drug resistance testing from Oxford Nanopore sequence data. Trop Med Int Health **2023**; 28:186–193.
