# Supplementary material for: A Real-Time Urine Tenofovir Assay Improves Drug Adherence Among People With HIV With Prior Virologic Failure in a Randomized Controlled Trial
Source: Clin Infect Dis. 2025 Jun 20;81(5):e352–9. doi: 10.1093/cid/ciaf337 (PMC12728291; doi:10.1093/cid/ciaf337)
Supplement: ciaf337_Supplementary_Data [file ciaf337_supplementary_data.zip › Supplement_4_HIV drug resistance table.docx]

**HIV drug resistance detected at 12-months study visit (study endpoint) among participants with and HIV viral load (HIV VL) of > 400 copies/mL**

| **PID** | **Regimen at the time** | **HIV VL** | **PI Major (Accessory)** | **NRTI** | **NNRTI** | **INSTI Major (Accessory)** |
| --- | --- | --- | --- | --- | --- | --- |
| P002 | TDF/FTC/LPV/r | 1146 | None | None | None | None |
| P004 | TDF/3TC/LPV/r | 990432 | None | None | K103KN | None |
| P011 | TDF/3TC/DTG | 20266 | None | None | K103N | None |
| P030 | TDF/FTC/LPV/r | 24234 | None | None | None | None |
| P033 | TDF/FTC/LPV/r | 15546 | None | A62AV | None | None |
| P034 | ABC/3TC/DTG | 80524 | None (L33F) | K65KR | None | None |
| P055 | TDF/3TC/DTG | 158163 | None | None | K103N, P225PH | None |
| P057 | TDF/FTC/ATZ/r | 8631 | M46MI, I50L, V82L, L90M (K20T) | K65KR, S68SN, M184V | P225H | None |
| P058 | TDF/FTC/ATZ/r | 3568 | None (G73S) | M184V | V108I, Y181C, H221Y, N348I | None |
| P065 | TDF/3TC/DTG | 742196 | None | None | K103N | None |
| P083 | TDF/3TC/DTG | 187548 | None | None | None | None |
| P091 | TDF/3TC/DTG | 5651 | None | None | None | None |
| P111 | TDF/3TC/DTG | 1362 | None | None | None | None |
| P117 | TDF/3TC/DTG | 174782 | None | None | E138A | None |
| P119 | TDF/3TC/DTG | 117194 | None | None | None | None |
| P126 | TDF/3TC/DTG | 982 | None | M184V | Y181C | None |
| P129 | TDF/3TC/DTG | 849 | None | None | None | None |
| P163 | TDF/3TC/DTG | 1755 | None | None | None | None |
| P130 | TDF/3TC/DTG | 452256 | V82L (L89T) | M184V | K103N, Y318F | None |
| P151 | TDF/3TC/DTG | 18317 | None | None | K103KN | None |
| P155 | TDF/3TC/DTG | 8983 | None | None | None | None |
| P162 | TDF/3TC/DTG | 2822 | None | None | None | None (Q95K) |
| P203 | TDF/3TC/DTG | 8785 | None (Q58E) | M184V | None | None |
